# Personal Experiences and Emotionality in Health-Related Knowledge Exchange in Internet Forums: A Randomized Controlled Field Experiment Comparing Responses to Facts Vs Personal Experiences

**DOI:** 10.2196/jmir.3766

**Published:** 2014-12-04

**Authors:** Joachim Kimmerle, Martina Bientzle, Ulrike Cress

**Affiliations:** ^1^Knowledge Media Research Center (KMRC)Knowledge Construction LabTübingenGermany

**Keywords:** personal experiences, emotionality, health, knowledge exchange, field experiment, Internet forums

## Abstract

**Background:**

On the Internet, people share personal experiences as well as facts and objective information. This also holds true for the exchange of health-related information in a variety of Internet forums. In online discussions about health topics, both fact-oriented and strongly personal contributions occur on a regular basis.

**Objective:**

In this field experiment, we examined in what way the particular type of contribution (ie, factual information vs personal experiences) has an impact on the subsequent communication in health-related Internet forums.

**Methods:**

For this purpose, we posted parallelized queries to 28 comparable Internet forums; queries were identical with regard to the information contained but included either fact-oriented descriptions or personal experiences related to measles vaccination. In the factual information condition, we posted queries to the forums that contained the neutral summary of a scientific article. In the personal experiences condition, we posted queries to the forums that contained the same information as in the first condition, but were framed as personal experiences

**Results:**

We found no evidence that personal experiences evoked more responses (mean 3.79, SD 3.91) from other members of the Internet forums than fact-oriented contributions (mean 2.14, SD 2.93, *t*_26_=0.126, *P*=.219). But personal experiences elicited emotional replies (mean 3.17, SD 1.29) from other users to a greater extent than fact-oriented contributions (mean 2.13, SD 1.29, *t*_81_=3.659, *P*<.001).

**Conclusions:**

We suggest that personal experiences elicited more emotional replies due to the process of emotional anchoring of people’s own style of communication. We recommend future studies should aim at testing the hypotheses with more general and with less emotionally charged topics, constructing different fact-oriented posts, and examining additional potential factors of influence such as personality factors or particular communication situations.

## Introduction

The Internet is an essential platform for sharing and exchanging information on a multitude of topics [1,2]. There is, however, great variety in the types of information that are shared on the Internet, ranging from purely fact-oriented information exchange to sharing very personal information [3-5]. People with a common interest in a particular topic may come together on the Internet as online communities in order to share information [6,7]. Platforms that are particularly relevant for information exchange are Internet forums [8,9]. These forums are virtual places where users can ask questions, express their opinions, and share their knowledge and experiences [10]. Participants in Internet forums can post messages that other users can pick up and reply to—instantly or at a later point in time. Many forums are organized and arranged according to certain topics or subtopics [8,10].

One subject area for which the Internet is an important platform for information search and information exchange is the field of health-related issues [11,12]. In the exchange of health-related information in pertinent Internet forums [5,10,13,14] and online health communities [15-18], there is also a wide range in the variety of contributions, with both fact-oriented information exchange and the transfer of personal information. Health-related Internet forums make it possible for everyone to make contributions, that is, ask or answer questions.

At the same time, this type of very open information exchange involves the risk that the reliability of health information is often very unclear [19-22]. Moreover, users who ask questions in an Internet forum are often not aware of the kinds of replies they can expect. They also cannot know under which circumstances their chances of receiving an answer are good or bad, if any answer at all [23]. Should they make their request as matter-of-factly, objectively, and neutrally as possible? Or do queries have a higher chance of eliciting an answer when they express personal concerns and experiences? And beyond the pure frequency of answers, what will reply posts look like then in terms of personalization and emotionalization? Will objective or personal queries elicit, in turn, answers that are respectively more neutral or more emotional?

Previous research has shown that people are very interested in personal information and care a lot about communication that includes personal and emotional aspects [4,24-26]. In particular, personal experience is considered a highly legitimate form of giving advice regarding health-related issues [26-28]. Accordingly, we state the following hypothesis: Hypothesis 1: users will reply more frequently to health-related queries that consist of personal experiences than to corresponding queries that contain fact-oriented information.

In addition, research has found that humans tend to imitate other people’s behavior [29] and the communication style of their communication partners [30-32]. In particular, people have a propensity to imitate expressions of emotion [33-35]. Moreover, emotionally expressive people were found to be more cooperative than people with a low level of emotional expressivity [36]. Accordingly, we state an additional hypothesis: Hypothesis 2: users will reply in a more emotional manner to health-related queries that consist of personal experiences than to corresponding queries that contain fact-oriented information.

## Methods

### Sample

We selected 28 Internet forums for this study. We took into consideration six different types of forums with regard to topic: 8 forums had a focus on health, 8 on family issues, 2 were sports forums, 2 dealt with naturopathy, 2 were vaccination critique forums, and 6 were general forums that dealt with a wide variety of topics including sports, health, etc.

From those considered, we selected only forums in which posts had occurred on the day of selection. Moreover, we ensured that the forums selected all had a comparable level of activity. A complete list of all 28 forums is included in Multimedia Appendix 1.

### Procedure

We applied a procedure that allowed for a randomized and controlled approach in combination with a high level of ecological validity. We created two experimental conditions: (1) factual information, and (2) personal experiences. In the factual information condition, we posted queries to the forums that contained the neutral summary of a scientific article. In the personal experiences condition, we posted queries to the forums that contained the same information as in the first condition, but were framed as personal experiences (see below). We randomly assigned an equal number of the different types of forums to the two experimental conditions, resulting in an allocation of 4 health forums, 4 family forums, 1 sports forum, 1 naturopathy forum, 1 vaccination critique forum, and 3 general forums in each condition.

We created user accounts for all forums on the same day and with the same gender-neutral user name. These accounts were used to post the queries in the respective forums on the identical day. We recorded the answers to these posts in the subsequent 4 weeks. In order to maintain absolute standardization of the experimental procedure, we did not reply to any answers or counter questions.

### Experimental Material

Both queries dealt with the topic of measles vaccination. We chose this topic because vaccination is a heavily debated issue about which many people have strong opinions and tend to express them on the Internet [37,38]. Accordingly, it appeared to be plausible that a substantial number of forum users would be willing to reply to such queries and express their opinion on this topic.

The query in the factual information condition was developed on the basis of an article published in the official journal of the German Medical Association [39]. This article reported the outbreak of measles in a medium-sized town and provided statistical descriptions of this epidemic. The query that was posted in this condition indicated that this user was looking for opinions about measles vaccination and had already gathered some information. This introduction was followed by a brief summary of the journal article including the complete reference. The provision of the reference was intended to emphasize the pure fact-orientation of this query and to make clear to the recipients that this information did not result from any kind of personal experience. This post consisted of 87 words.

The query in the personal experiences condition also stated that the user was looking for opinions about measles vaccination and had previously gathered some information. This introduction was followed by the same information as in the previous condition (regarding the age of the children concerned and the symptoms), but was framed in terms of personal experience with the outbreak of measles in a day-care center. This post did not refer to the journal article and consisted of 69 words.

### Measures

We counted the number of replies and captured the average number of replies to the posts in the two experimental conditions in order to test Hypothesis 1. In addition, two independent raters evaluated the emotionality of the replies on 5-point Likert scales ranging from 1 (neutral) to 5 (very emotional), in order to allow for examining Hypothesis 2. Those raters were blind to the respective experimental condition and to the general research questions, and reached consensus on their final rating in cases of disagreement.

The following reply is an example of a very emotional statement:

For me, measles vaccination is obligatory and I was really glad that my little kid could finally be vaccinated now in May! Because I am extremely frightened of the long-term consequences and I personally rather put up with the very unlikely vaccination adverse effects than running the risk of a measles infection. Once in the hospital I had to care for a child suffering from meningitis, which died then, and this experience has probably affected me …

This reply, in contrast, is an example of an emotionally neutral statement:

Yes, measles are contagious, yes, children can fall ill with them, and yes, one can be vaccinated against them, which is worthwhile, because measles are known for having an impact on fertility.

### Statistical Analysis

We compared the number of replies within the different types of forums using chi-square tests. In order to test Hypothesis 1, we compared the number of replies with a chi-square test and conducted an independent samples *t* test with the average number of replies as dependent variable. To take the distribution of this variable into account, we additionally conducted a Mann-Whitney *U* test and a Mood’s median test. For testing Hypothesis 2, we conducted an independent samples *t* test with emotionality of the replies as dependent variable.

### Ethical Considerations

Since this study used information posted in open online forums, contributions were considered as belonging to the public domain [40,41]. The posts of all users in all forums could be openly read by everybody; usernames and passwords were only required for posting. So the behavior of research participants did not occur in a private context [42]. In line with the requirements of the local ethics committee at the Knowledge Media Research Center (Tübingen, Germany), the dignity and integrity of participants were not violated in any way in this study. We did not give medical advice to the forum users. We did not provide any medically questionable or incorrect information in the online forums.

We made sure that our queries did not confront the users with any kind of information that they would not otherwise encounter in Internet forums or many other communication situations. Thus, the forum users were not taken advantage of in any respect; there was absolutely no infringement of the users’ rights. In order to protect the individual user’s personal or online identity, we do not provide information about the names or online identities of contributors in this article. In order to avoid an individual’s identity being traced by searching for the quoted phrases, we use only English translations of direct quotes [42,43].

## Results

### Number of Replies

There was a wide range in the number of replies among the different Internet forums: the number of replies per forum varied from 0 to 13. The most replies were found in the 8 family forums (a total of n=37; mean 4.63, SD 3.89) compared to the other 20 forums (a total of n=46; mean 2.30, SD 3.18), as indicated by a chi-square test (χ^2^_10_=22.12, *P*=.015). The other forum types did not differ among each other in the frequency of replies, all *P*s>.370.

Regarding Hypothesis 1, there appeared to be more replies (n=53) to the personal experiences than to the factual information (n=30), with a median of 2 in the personal experiences condition and a median of 1 in the factual information condition. But the two conditions did not differ significantly from each other when we compared the number of replies with a chi-square test (χ^2^_10_=8.40, *P*=.590).

The same finding occurred when we conducted an independent samples *t* test with the average number of replies as dependent variable (*t*_26_=1.26, *P*=.219). In the factual information condition, the posts received on average 2.14 (SD 2.93) replies per forum. In the personal experiences condition, we found 3.79 (SD 3.91) replies.

We also conducted further nonparametric tests, but both a Mann-Whitney *U* test (*P*=.178) and a Mood’s median test (*P*=.257) confirmed that the difference was non-significant.

### Emotionality of Replies

As expected in Hypothesis 2, in a *t* test the replies of the other forum users were significantly more emotional in the personal experiences condition (mean 3.17, SD 1.29) than the answers in the factual information condition (mean 2.13, SD 1.29; *t*_81_=3.66, *P*<.001).

## Discussion

### Principal Findings

Contrary to our initial assumptions, personal experiences did not elicit more replies than factual information. This absence of a significant effect was probably due to the very large variance of replies in the different forums. It might also be the case that forum users tended to regard factual information and personal experience as equivalent. This interpretation would be consistent with previous findings that forum users in health-related contexts do not adequately distinguish between verifiable and non-verifiable information [10,16,21,22].

The key finding of this field experiment is that answers to a post containing personal experiences were significantly more emotional than replies to factual information. Reasons for this could be that inquirers who reported private information were perceived as more personable or that the recipients of the initial posts felt addressed on a personal level and, accordingly, replied on the same level. This behavior might be explained by effects of anchoring and adjusting [44-46], in that participants used the initial post as an emotional anchor to which they adjusted their own style of communication.

### Limitations and Future Work

The finding that reply rates were rather low overall could be due to the topic we selected. Potentially, and contrary to our expectations that people would feel strongly about the topic, the subject area of measles vaccination is only important to a limited group of people, that is, people who have young children themselves. It may be a rather abstract or even an irrelevant topic for childless people, such as singles or adolescents. This interpretation is supported by the finding that the family forums were the most active ones regarding our queries, compared to forums concerned with other topics. In addition, most answers implied that they were written by people who had personal experience with this topic. This might also indicate that vaccination is in fact an emotionally charged topic for many people, a circumstance that may have had an impact on their response behavior. Future studies should aim to test the hypotheses with different, that is, more general topics with a broader base of interest and with a less emotionally charged subject matter.

Another limitation of our study is that the queries in the factual information condition might have been perceived to be quite uncommon or peculiar, because it is rather seldom that posts in Internet forums include a reference to a journal article. Even though the provision of this reference was intended to underline the fact-orientation of the post, it is not clear how the forum users perceived this information. Future research should try to find ways to construct fact-oriented posts that do not appear to be peculiar to other forum users.

In addition, the emotionality of the reply posts was only evaluated by two independent raters. Future studies might consider applying additional assessment methods, such as identifying key words of emotionality in the reply post of the forum users [47,48].

Finally, it might be worthwhile for further research to examine additional potential factors of influence that may have an impact on the way people express their answers. This may include certain personality factors, such as social value orientation [49], agreeableness [50], or people’s need for self-presentation and self-esteem [51,52]. In addition, these factors of influence may also include aspects of the communication situation, such as anonymity [53], identifiability [54-56], or the synchronicity [57-59] of information exchange. The current study represents only a beginning to potential research into what impact particular kinds of contributions have on the subsequent exchanges of health-related information in Internet forums.

## Multimedia Appendix 1

List of Internet forums.
