# Supplementary material for: Personal Experiences and Emotionality in Health-Related Knowledge Exchange in Internet Forums: A Randomized Controlled Field Experiment Comparing Responses to Facts Vs Personal Experiences
Source: J Med Internet Res. 2014 Dec 4;16(12):e277. doi: 10.2196/jmir.3766 (PMC4275470; doi:10.2196/jmir.3766)
Supplement: Supplementary file 1 [file jmir_v16i12e277_app1.pdf]

Appendix 1

List of all 28 Internet forums:

1. http://www.frag-dich-gesund.de/forum/
2. http://www.gesundheit.de/forum
3. http://www.lisa-freundeskreis.de/magazin/gesundheit/
4. http://www.medizin-forum.de/phpbb/viewtopic.php?f=51&t=113928
5. http://www.med1.de/Forum/
6. http://www.mein-gesundheitsforum.de/g_forum/index.php
7. http://www.onmeda.de/foren/
8. http://bfriends.brigitte.de/foren/gesundheit/
9. http://www.impfschutzverband.de/phorum5/
10. http://www.impfschaden.info/de/impfforum.html
11. http://www.babyclub.de/community/foren/
12. http://www.netmoms.de/gruppen/kategorie/gesundheit
13. http://www.familienmafia.de/entwicklungsforum
14. http://www.elternforen.com/
15. http://www.babyforum.de/
16. http://www.naturwindeln.de/forum/
17. http://www.hochzeitsplaza.de/hochzeits-forum/
18. http://www.urbia.de/forum/
19. http://www.gofeminin.de/
20. http://www.gutefrage.net/
21. http://www.yazio.de/fragen
22. http://www.allround-forum.net
23. http://www.allround-talk-forum.com/
24. http://www.allgemein-forum.com/
25. http://www.natur-forum.de/forum/
26. http://www.cysticus.de/naturheilkunde-forum/
27. http://www.sportsuche.info/forum/
28. http://www.sportlerfrage.net
